# Anti-CD19 Chimeric Antigen Receptor T Cells in Combination With Nivolumab Are Safe and Effective Against Relapsed/Refractory B-Cell Non-hodgkin Lymphoma

**DOI:** 10.3389/fonc.2019.00767

**Published:** 2019-08-19

**Authors:** Yaqing Cao, Wenyi Lu, Rui Sun, Xin Jin, Lin Cheng, Xiaoyuan He, Luqiao Wang, Ting Yuan, Cuicui Lyu, Mingfeng Zhao

**Affiliations:** ^1^The First Central Clinical College of Tianjin Medical University, Tianjin, China; ^2^Department of Hematology, Tianjin First Central Hospital, Tianjin, China; ^3^Nankai University School of Medicine, Tianjin, China

**Keywords:** B-cell non-Hodgkin lymphoma (B-NHL), anti-CD19 chimeric antigen receptor T cells, immune check point blocade, combination, safe and effective

## Abstract

Chimeric antigen receptor (CAR) T cells are emerging as a novel treatment for patients with refractory/relapsed B-cell non-Hodgkin lymphoma (B-NHL), and combination with PD1 inhibitors may further improve the efficacy of anti-CD19 CAR (CD19 CAR)-T cells in the treatment of lymphomas. In a single-center study, we evaluated the safety and efficacy of a combination therapy with CD19 CAR-T cells and an anti-PD-1 antibody (nivolumab) in patients with relapsed/refractory B-NHL. A total of 11 patients with refractory/relapsed B-NHL were recruited and subsequently received CD19 CAR-T cells and nivolumab. The primary end points were safety and feasibility. The infusions were safe, and no dose-limiting toxicities occurred. Grade 1 or 2 cytokine release syndrome (CRS) was observed in 25% (3/11) and 50% (6/11) of the patients, respectively, and only one patient (1/11) experienced neurotoxicity. The objective response rate (ORR) and complete response (CR) rate were 81.81% (9/11) and 45.45% (5/11), respectively. The median follow-up time was 6 (1~15) months. The median progression-free survival (PFS) time was 6 months (1~14 months), and 3 patients continued to have a response at the time of this writing. Our study demonstrated that the combination of CD19 CAR-T cells and nivolumab was feasible and safe and mediated potent anti-lymphoma activity, which should be examined further in prospective clinical trials in refractory/relapsed B-NHL.

## Introduction

B-cell non-Hodgkin lymphoma (B-NHL) is a hematological malignancy with high heterogeneity and includes diffuse large B-cell lymphoma (DLBCL), mantle cell lymphoma (MCL), and follicular lymphoma (FL). With the development of treatment strategies, especially with the prevalence of the anti-CD20 monoclonal antibody rituximab, the remission rate of B-NHL has been elevated to a large degree. However, resistance and recurrence remain to be resolved. Recently, chimeric antigen receptor (CAR) T cells have emerged as a novel treatment modality for these patients (1, 2). CAR-T cells, which are genetically modified to express a specific CAR, can specifically recognize target antigens and kill target tumor cells. CD19 is specifically expressed on the surface of B-lymphocytes at different stages of differentiation, and more than 95% of B-cell lymphomas express the CD19 antigen. The administration of CAR-T cells that recognize CD19 can achieve therapeutic efficacy in B-lymphocyte tumors. However, unlike the favorable results in B-cell lymphocytic leukemia, the clinical benefit of anti-CD19 CAR (CD19 CAR)-T cell therapy in lymphoma is limited, partially due to the development of an immunosuppressive tumor microenvironment (3, 4). To bolster the potency of CAR-T cells, the modulation of the immunosuppressive tumor microenvironment with immune checkpoint therapy is a promising strategy (4).

Immune checkpoint therapy is a treatment approach that enhances the antitumor immune response of T cells by blocking the immunosuppressive pathways activated by cancer cells. The PD-1/PD-L1 axis is a key immune checkpoint that suppresses T cell-mediated immune responses. The expression of PD-1 in CD19 CAR-T cells is increased after infusion into patients with B-cell malignancies, and PD-1 disruption may enhance the effectiveness of CAR-T cell treatment (5–7). A case report further revealed that PD-1 blockade can be effective against refractory lymphoma that fails to respond to CAR-T cell therapy, which may be due to a new round expansion of the CD19 CAR-T cells (8). Although CAR-T cell therapy in combination with PD-1 blockade is a potential treatment modality, this rational combinatorial strategy may result in T cell over activation, eventually leading to enhanced toxicities such as cytokine release syndrome (CRS) or neurological damage. There is currently no public report evaluating the clinical outcome of CD19 CAR-T cells in combination with anti-PD-1 antibody therapy to treat B-NHL. The aim of this study was to evaluate the feasibility, safety, and efficacy of CD19 CAR-T cell treatment combined with PD-1 inhibition via nivolumab in patients with relapsed/refractory B-NHL.

## Methods

### Patients and Study Design

This study was a retrospective cohort study of 11 consecutive patients with relapsed/refractory lymphoma [defined as progressive or stable disease as the best response to the most recent chemotherapy regimen or disease progression or relapse within 12 months after autologous stem cell transplantation (ASCT)] (1) who received an infusion of autologous CD19 CAR-T cells between May 1st, 2017, and November 20th, 2018. Among them, all 11 patients received the anti-PD-1 antibody nivolumab after an infusion of CD19 CAR-T cells. Eligible patients met all of the following criteria: (1) histologically confirmed CD19-positive B-cell lymphoma diagnosed on the basis of the 2016 World Health Organization guidelines; (2) refractory disease, which was defined as progressive or stable disease as the best response to the most recent chemotherapy regimen or disease progression or relapse within 12 months after ASCT (1); (3) at least one measurable lesion according to the revised International Working Group (IWG) response criteria; (4) no evidence of central nervous system (CNS) lymphoma by magnetic resonance imaging; (5) aged ≥18 years and ≤ 80 years with an ECOG score ≤ 2, an absolute neutrophil count ≥1,000/mL, and a platelet count ≥50,000/ml. Key exclusion criteria included a history of malignancy, a history of allogeneic hematopoietic stem cell transplantation and severe renal, hepatic, and cardiac dysfunction.

The study was approved by the institutional review board at Tianjin First Center Hospital and was conducted in accordance with the Good Clinical Practice guidelines of the International Conference on Harmonization. All the patients were recruited into our CAR-T cell clinical trial for relapsed or refractory B-cell lymphoma (ChiCTR-ONN-16009862, ChiCTR1800019288). The patients were informed about the potential clinical benefits and potential adverse events (AEs) of the treatment regimen including CD19 CAR-T cells and nivolumab and provided written informed consent. Ethical approval for this study was granted by the Tianjin First Central Hospital Medical Ethics Committee.

### CAR T Cell Manufacturing

CD3+ T cells were separated from peripheral blood mononuclear cells (PBMCs) with CD3 immunomagnetic beads (Miltenyi) on day 1. The T cells were expanded with CD3/CD28 stimulating beads (Gibco) and IL-2 overnight and then transduced with a lentiviral vector containing a CAR with a CD3-zeta domain to provide a T cell activation signal and a 4-1BB (CD137) domain to provide a co-stimulatory signal (Creative Biolab). Next, the transfection efficiency was detected 5 days after transfection, and the cells were then expanded for another 5 days until the numbers were sufficient for infusion.

### Treatment Plan

The disease burdens of all enrolled patients were assessed at baseline via computed tomography (CT)/positron emission tomography (PET) imaging.

All patients received low-dose conditioning chemotherapy with concurrent cyclophosphamide (500 mg/m^2^) and fludarabine (30 mg/m^2^) administration for 3 days followed by CD19 CAR-T cell infusion. The patients were given 3 mg/kg PD-1 inhibitor (Opdivo) in a single dose on the 3rd day after CD19 CAR-T cell infusion. Patients with CRS received symptomatic treatment. Laboratory testing was performed on day−3 and day 3, and during weeks 1, 2, 3, 4, 5, and 6 after infusion for all patients and included evaluating the expansion of the CD19 CAR-T cells, the level of PD-1 expression, the subgroups of lymphocytes and the levels of cytokines.

### Clinical Response Assessment

Response to therapy was evaluated by PET-CT or CT imaging, and bone marrow was evaluated 6 weeks after the initial infusion by standard pathological testing when involved according to Revised IWG Response Criteria for Malignant Lymphoma (9).

### Statistical Analysis

The primary end points were safety and feasibility. The secondary end points included the objective response rate (ORR), the duration of response, the complete response (CR) rate, the expansion and persistence of the CD19 CAR-T cells and the serum cytokine levels. CRS was graded according to the criteria of Lee et al. (10). We used the National Cancer Institute Common Terminology Criteria for Adverse Events version 4.03 to grade the CRS symptoms and neurological events along with other AEs.

## Results

### Patient Characteristics

Eleven patients were enrolled in this trial. Table 1 shows the clinical and disease specific characteristics of the patients. The median age was 65 years (range, 26~75 years). The 11 patients included 1 patient with Burkitt's lymphoma, and the remaining were diagnosed with DLBCL. Five of the 11 patients had primary refractory disease (no achievement of CR with 4 cycles of first-line chemoradiotherapy); the remaining six patients had disease that relapsed and was refractory to the most recent prior therapy. The disease statuses of all 11 patients were classified as progression before CD19 CAR-T cell infusion.

**Table 1:** The clinical and disease specific characteristics of the patients.

**Patient no**.	**Age (years)**	**Sex**	**Diagnosis/Stage**	**Disease status**	Extranodal sites**	**Prior treatment**		
						**Chemotherapy regimens#**	**ASCT**	**Others**
1	69	F	DLBCL/IV	Primary refractory*	Muscle	15*CHOP/GDP/R-GDP	N	
2	65	M	DLBCL/IV	Relapsed		4*R-CHOP	N	
3	49	M	DLBCL/IV	Relapsed		4*R-CHOP	Y	
4	75	F	DLBCL/III	Primary refractory	Kidney, pancreas	4*R-CHOP	N	
5	70	M	DLBCL/IV	Relapsed	Ileocecus	6*R-CHOP/R-MINE	N	
6	62	F	DLBCL/IV	Primary refractory	Uterus	5*R-CHOP/R-COP	N	
7	39	F	DLBCL/IV	Relapsed	Liver	FC/R-COPE/R-CHOPE/R-GEMOX	Y	
8	70	F	DLBCL/III	Relapsed	Bone marrow	4*R-CHOP/R-EPOCH	N	Lenalidomide
9	26	M	Burkitt's lymphoma/IV	Primary refractory	Ileocecus	2*R-HyperCVAD/RMA/CODOX-M	N	Radiotherapy
10	67	M	DLBCL/IV	Relapsed	Bone Marrow	3*R-EDOCH/R-CHOP/Hyper-CVAD/DHAP/GDP/GDPE	N	
11	40	M	DLBCL/IV	Primary refractory		3*R-CHOPE/2 *R-CHOP/3*R-DHAP	N	Radiotherapy

^#^*ASCT, autologous stem cell transplantation; CHOP, cyclophosphamide, adriamycin, vincristine, prednisone; GDP, gemcitabine, dexamethasone, cisplatin; GEMOX, gemcitabine, oxaliplatin; ICE, ifosfamide, carboplatin, etoposide; R, rituximab; EPOCH, etoposide, prednisone, vincristine, cyclophosphamide, doxorubicin; CVAD, cyclophosphamide,mesna, pharmorubicin, vindesine, dexamethasone. DAHP, cisplatin, Cytarabine, dexamethasone. GDPE, gemcitabine, dexamethasone, cisplatin; etoposide.*

* Primary refractory: no achievement of CR with 4 cycles of first-line chemoradiotherapy.

** *The results obtained according to PET/CT*.

The patients received a median of 8 ×106/kg (range 5–11 ×10^6^/kg) CD19 CAR-T cells in total during infusion (Table 2). All patients were infused with one dose of 3 mg/kg nivolumab 3 days after CD19 CAR-T cell infusion.

**Table 2:** Patient response and toxicity.

**Patient no**.	**Disease status at study entry**	**Conditioning regimen before T cell infusion**	**No. of CAR-positive T cells infused (106/kg)**	**Response**		**CRS grade**	**Grade 3 toxicities (excluding CRS)**
				**Type**	**Duration**		
1	PD	FC	8	PR	5	1	None
2	PD	FC	10	PR	5	2	None
3	PD	FC	5	PR	6	2	Febrile neutropenia, anemia, neutropenia, thrombocytopenia
4	PD	FC	11	NR	/	None	None
5	PD	FC	6	CR	14+	1	None
6	PD	FC	11	CR	13+	None	None
7	PD	FC	8	CR	9	2	Gamma glutamyl transpeptidase level increased, febrile neutropenia, anemia, neutropenia, thrombocytopenia
8	PD	FC	8	CR	6+	1	None
9	PD	FC	11	PR	1	2	Gamma glutamyl transpeptidase level increased
10	PD	FC	6	CR	1	2	Heart failure, thrombocytopenia
11	PD	FC	5	NR	/	2	Febrile neutropenia, neutropenia

### Safety

CRS occurred in nine patients, and all the cases were of low grade (3 grade 1 cases and 6 grade 2 cases). One patient's symptoms were relieved with corticosteroids, and the other eight patients received only symptomatic treatment (administration of non-steroidal anti-inflammatory drugs (NSAIDS) to relieve symptoms) for the management of CRS. There was one episode of neurotoxicity in Patient 3, with the development of tremors that resolved with symptomatic treatment on day 7 after presentation.

Grade 3 or 4 AEs were reported in 45.5% of all treated patients (Table 2). Nearly all patients had cytopenia, including neutropenia, thrombocytopenia, or anemia, to different degrees resulting from chemotherapy, and febrile syndrome occurred 0.5–2 h after CD19 CAR-T cell infusion and self-recovered overnight. The most common toxicities were fever (72%), fatigue (54.6%), and nausea (27.3%).

Overall, all toxicities were manageable and reversible. No serious immune-mediated AEs, such as nephritis or hepatitis, were observed in any of the treated patients with extended follow-up. No deaths were attributable to treatment-related toxicity.

### Efficacy

**Figure 1:**
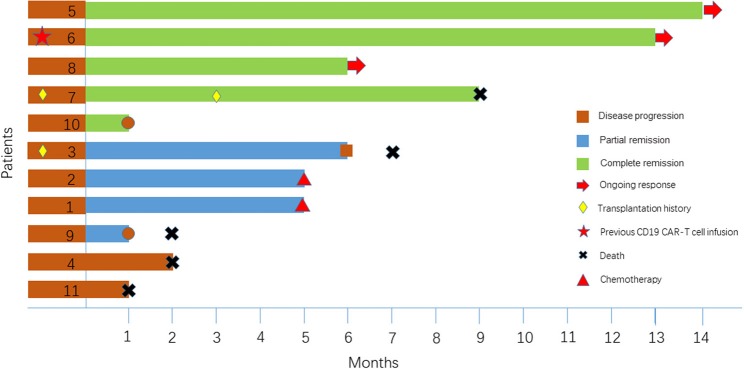
Clinical response and response duration in 11 enrolled patients.

Of the 11 patients, 9 (81.8%) achieved an objective response, and 45.5% (5/11) achieved a CR (Table 1). All patients were observed for at least 1 month to monitor response (Figure 1). The median PFS time was 6 months (1~14 months), and 3 patients continued to have a response at the time of this writing. Patient 4 and Patient 11 had no response to the CD19 CAR-T cell infusion and anti-PD-1 antibody therapy and had a poor prognosis (indicated by BCL-2/C-myc double-positive expression, P53 deficiency and a large tumor bulk).

**Figure 2:**
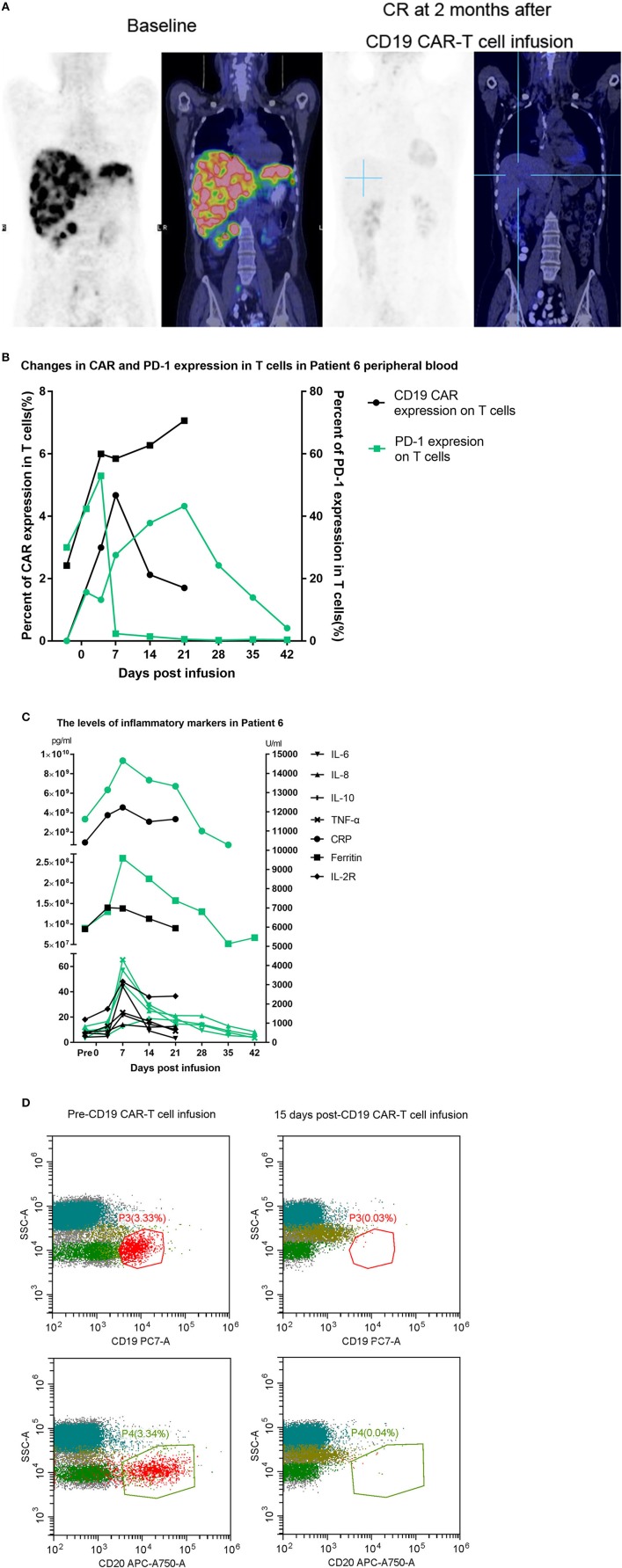
**(A)** Whole-body scans showing lymphoma lesions in Patient 7 detected by PET/CT before and after CAR-T cell therapy. **(B,C)** The black line represents the previous CD19 CAR-T cell infusion, and the green line represents the later combination treatment. **(D)** Patient 8 had a bone marrow involvement. These malignant B-cell disappeared after CD19 CAR-T infusion.

Two patients, Patient 3 and Patient 7, had disease recurrence after ASCT. Patient 7 relapsed within 2 months after ASCT, and she then received CD19 CAR-T cell therapy and achieved a CR. Three months later, she underwent allogeneic hematopoietic stem cell transplantation. She had an ongoing CR for 7 months after transplantation but died of a serious infection in the 7th month. Figure 2A indicates that the enlarged diffuse multiple lesions in the liver disappeared. Similar to Patient 7, Patient 3 underwent ASCT but relapsed 22 months later. He then achieved a partial response (PR) within 2 months after infusion of the CAR-T cells. Nivolumab was administered to achieve a better result and decreased the expression of PD-1 after infusion. However, no significant expansion of the CD19 CAR-T cells was observed. Finally, he maintained the PR for 5 months but ultimately died due to disease progression.

Patient 6 had previously received an infusion of CD19 CAR-T cells but achieved only a PR 1 month later, and few CD19 CAR-T cells could be detected in her peripheral blood. One month later, the proportion of CAR-T cells expressing PD-1 reached 52.23%. Considering the limited proliferation and antitumor function of the CD19 CAR-T cells, we assumed that the expansion of the CD19 CAR-T cells was inhibited by the PD-1/PD-L1 interaction, which led to treatment failure. She finally achieved a CR after a second CD19 CAR-T cell infusion accompanied by nivolumab. Although we detected that the expression of PD-1 declined toward 0, we did not detect a significant increase in the CD19 CAR-T cell number (Figure 2B) relative to the number during the expansion after the first infusion. However, there was relatively stronger cytokine release, and the persistence of the CD19 CAR-T cells was more durable (Figures 2B,C).

Patient 8 was infused with nivolumab for a year and maintained a CR until relapsing 1 month before being enrolled. PET-CT imaging indicated that there was disease progression, and flow cytometry (FCM) analysis suggested that the bone marrow was involved. Very few CD19-positive cells were detected 15 days after CD19 CAR-T cell infusion in the bone marrow (Figure 2D). Patient 8 obtained a CR 1 month later.

### CAR-T Cell Expansion and Biomarker Analysis

**Figure 3:**
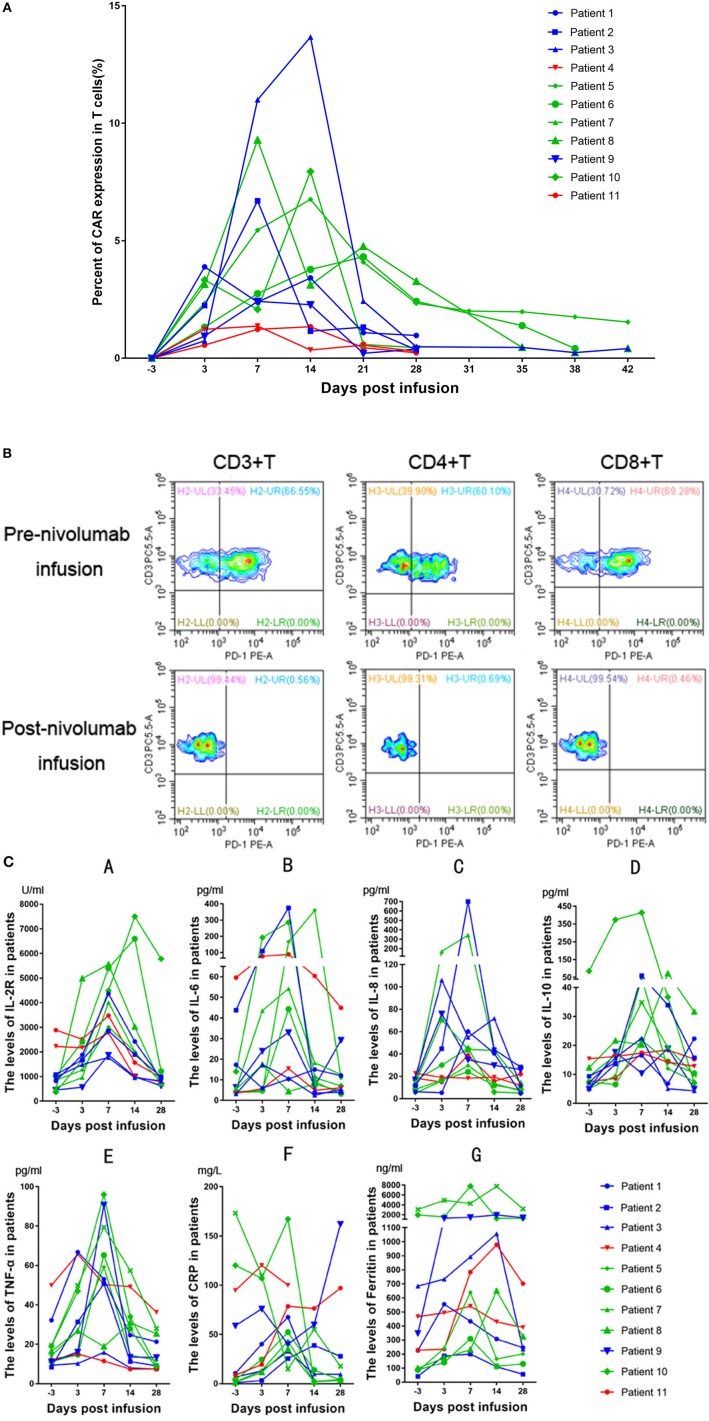
**(A)** Peripheral blood CD19 CAR-T cells in patients receiving treatment. Patients who obtained a CR are represented in green, patients who obtained a PR are represented in blue, and patients who had no response are represented in red. **(B)** Patient 8 had a bone marrow involvement. These malignant B-cell disappeared after CD19CAR-T infusion. **(C)** Changes in inflammatory marker levels in patients after CD19 CAR-T cell and nivolumab infusion. The green lines, blue lines and red lines represent patients having obtained a CR, PR, or NR, respectively. A, IL-2R. B, IL-6. C, IL-8. D, IL-10. E, TNF-α. F, CRP. G, Ferritin.

Peak expansion of CAR-T cells occurred within the first 7–14 days after infusion. The remaining CD19 CAR-T cells were detectable at low levels by flow cytometry analysis for up to 1 month in the peripheral blood of all patients with an ongoing CR (Figure 3A). As we previously hypothesized, the flow cytometry results showed that the expression of PD-1 declined sharply to a level that could not be detected (Figure 3B). In the patients, the plasma levels of cytokines, including IL2, IL4, IL6, IL8, IL10, TNF-α, and IFN-γ, as well as the levels of CRP were analyzed at serial time points before and after CD19 CAR-T cell infusion. There were significant increases in the levels of IL-2R, IL-6, IL-8, IL-10, TNF-α, and ferritin within 2 weeks (Figure 3C). However, no significant differences in CRP levels were observed among the patients with different clinical responses. In the group of patients who had no response (NR), the expansion of CD19 CAR-T cells and the cytokine levels tended to be lower and flatter.

## Discussion

Relapsed/refractory B-NHL comprises a heterogeneous patient population, and outcomes in these patients are particularly poor (11). Multicenter clinical trials for relapsed or refractory DLBCL have showed encouraging preliminary data, with CR rates ranging from 33 to 57% (12, 13). Despite some cheering therapeutic successes, CAR-T cells, similar to endogenous T cells, demonstrate susceptible to inhibitory immune checkpoints present in the tumor microenvironment, such as the PD-1/PD-L1 axis (4). In the current study, we evaluated the safety and efficacy of CD19 CAR-T cells combined with a PD-1 antibody in relapsed/refractory B-NHL. Our study demonstrated that (1) CD19 CAR-T cell treatment (5–11 ×10^6^ cells/kg) followed by nivolumab at a dose of 3 mg/kg is well tolerated with manageable toxicity; (2) PD-1 expression on T cells may increase after CAR-T cell infusion but decreases significantly after PD-1 blockade; and (3) further clinical studies should be performed to identify whether the combination of CD19 CAR-T cells and an anti-PD-1 antibody improves the prognosis of B-NHL.

Immune checkpoints include programmed death molecules and their ligands (PD-1/PD-L1), lymphocyte activation gene 3 (LAG-3), cytolytic T lymphocyte-associated antigen 4 (CTLA-4), B/T lymphocyte attenuator (BTLA), T cell membrane protein 3 (TIM-3), and adenosine A2a receptor (A2aR). Among these checkpoints, PD-1/PD-L1 signaling is the most commonly targeted. CAR-T cells, like their endogenous counterparts, acquire a differentiated and exhausted phenotype associated with increased expression of PD-1^C^. Significant activation-induced cell death of CAR-T cells, which was PD-1 dependent, has been observed after repeated antigen stimulation. The results of preclinical experiments in mouse models have demonstrated that combining CAR-T cell therapy with PD-1 blockade can improve CAR-T cell activity and promote increased tumor cell death. However, clinical experience employing the combination of CAR-T cells and immune checkpoint blockade is still in its early stages (4).

A case report revealed that PD-1 inhibition can be effective against DLBCL that fails to respond to CD19 CAR-T cell therapy and augments the expansion of CD19 CAR-T cells (8). Furthermore, this research group that published the case report recently conducted a phase I/II clinical trial of pembrolizumab in patients with B-NHL expressing CD19 that was relapsed/refractory after CD19 CAR-T cell therapy. The ORR after CD19 CAR-T cell therapy followed by nivolumab infusion was 27% [1 CR; 2 PR]; 1 patient had stable disease (SD), and 7 patients had progressive disease (PD) (14). A case series reported that CAR T cell administration in combination with lymphodepletion and PD-1 inhibition in patients with neuroblastoma (NB) did not further enhance expansion or persistence (15), which was consistent with our results. This article indicated that although CRS after salvage therapy were observed only in the CAR-T cell infusion combined with anti-PD-1 antibody treatment cohort, no conclusions could yet be drawn from such limited observations in such a heterogeneous group of patients. The authors assumed that the beneficial effects of checkpoint blockade on CAR-T cell persistence and expansion may depend on the timing and duration of the PD-1 inhibition or may require the presence of a greater number of tumor neoantigens (stimulating CAR-T cells through their native TCRs) than are present in most pediatric malignancies, including NB (15). Thus, the clinical response reflected the combined effects of the two treatments. Our study, in which we selected 3 days after CD19 CAR-T cell infusion as the time to inhibit PD-1, showed that the ORR and CR rates in the CAR-T cells combined with nivolumab group were 81.81 and 45.45%, respectively. Two patients relapsed within 12 months after ASCT patients have satisfying efficacy (1CR, 1PR), which were better than the primary refractory (2CR, 1PR, 2NR). We estimated that two interference factor are responsible. Firstly, the number is too small to interpret precisely. Next, the two NR patients deteriorated rapidly. Or that previous ASCT endowed patients with a relative normal immune microenvironment, friendly to the CD19 CAR-T cells. Furthermore, the expression of PD-1 on T cells was significantly decreased after nivolumab treatment, and inflammatory cytokine secretion was dramatically elevated with the combination treatment, which may indirectly reflect the enhanced antitumor function of the CAR-T cells. However, the number of CD19 CAR-T cells in our patients did not seem to be higher than the number measured in the patients in our center who received CD19 CAR-T cell infusion alone (data not shown). And the effect of the recruited patients seems to be no superior to than the effect of the patients in our center who received CD19 CAR-T cell infusion alone (CR:5/11, PR:3/11, SD:1/11, NR:2/11; Supplementary Tables 1, 2). However, patients treated with CD19 CAR-T combined with nivolumab had more adverse prognostic factors than patients treated with CD19 CAR-T alone, so the efficacy was not comparable. Nevertheless, our preliminary result showed that CD19 CAR-T combined with nivolumab had a relatively satisfactory effect for the patients with adverse prognosis and no more toxicities.

It should be noted that nivolumab has anti-tumor activity independent of CAR-T cells. Malignant cells can escape T cell-mediated cellular cytotoxicity by utilizing the inhibitory PD-1/PD-L1 immune checkpoint (16). The expression of PD-L1 is associated with poor overall survival in patients with DLBCL, and therapeutic antibodies that block PD-1 on T cells induce durable clinical responses against lymphoma (17, 18). We performed immunohistochemistry with 9 available pathological section and got the relevant PD-L1 expression (Data not shown). We find that an absence of response or an early relapse have no relationship with the expression of PD-L1 in tumor tissues. And the blockade of PD-1 does not brought benefits for all patients with a high PD-L1 expression (Patient 5, 6, 8, 9, 11> 50%). Similar conditions also occurred in patients with a relative high PD-1 expression. Larger number of patients need to be recruited for further study. Or that there is some other immunosuppression axis such as TIM-3, LAG-3 limited the function of CD19 CAR-T. For PD-1 blockade with nivolumab has been demonstrated to result in response rates as high as 87% in relapsed/refractory Hodgkin lymphoma, 36% in DLBCL and 40% in follicular lymphoma (18, 19). These data could also explain the favorable response of B-NHL to CAR-T cell and nivolumab treatment in our study. However, a recent clinical study indicated that in patients with DLBCL who are ineligible for autologous hematopoietic stem cell transplantation (auto-HSCT) or who experience failure after auto-HSCT, nivolumab monotherapy is safe but has a low ORR (20). Namely, nivolumab monotherapy cannot be selected as a salvage therapy for B-cell lymphoma. Patient 8 received nivolumab infusion and maintained a CR for a year but experienced disease progression before being recruited into our study. A significant expansion of CD19 CAR-T cells in the peripheral blood was detected after combined therapy. We hypothesize that anti-PD-1 antibody monotherapy can endow T cells with a powerful antitumor ability. Although autologous T cells have limited specific antitumor effects, an anti-PD-1 antibody accompanied by an infusion of CD19 CAR-T cells, which can specifically lyse tumor cells, can increase antitumor effectiveness.

However, treatment with checkpoint inhibitors brings about potentially toxicities and is associated with high costs. Thus, the safety of this combination treatment is an important issue. In our study, we used only one 3 mg/kg dose of nivolumab, which minimized the cost while producing a favorable effect. Most importantly, we did not find enhanced AEs, including CRS and neurotoxicity, with the combination treatment. The low-grade CRS and neurotoxicity may be associated with close monitoring and early intervention after CAR-T cell treatment. On the other hand, this approach is also the reason that our ORR and CR rates were lower than those seen with CD19 CAR-T cell therapy in some studies conducted at single institutions. No serious infection occurred as a result of the combined administration, for only Patient 7 developed infection after ASCT and died finally. Rates of immune-mediated AEs were low in all patients with extended follow-up. The only informed immune-mediated AEs was mild diarrhea after infusion (Patient 5).

PD-1 blockade can refuel exhausted T cells by disturbing the interaction between PD-1 and PD-L1 on tumors or other immune-suppressive cells. This could support the long-lasting antitumor ability of CD19 CAR-T. And in patients with high tumor burden, CD19 CAR-T alone is too weak to reduce the mass in some degree, exhausted by the immunosuppress microenvironment. A combined therapy may do favor to a good outcome. As well as the patients with high PD-1 expression on T cells or high PD-L1 expression on tumor tissues. For that just a relaxation for CD19 CAR-T is a favorable factor. And the most important, when companied by adverse prognostic factors, such as double-hit or triple-hit, combined therapy may endow CD19 CART more power to reduce tumor.

Some limitations still exists in our study. A single-center study and small number of patients recruited made that reasonable statistics analysis unavailable. Although ASCT is the standard of therapy for patients with relapsed/refractory DLBCL, it is marked by high recurrence rates and serious AEs. Whether combining CD19-CART with anti-PD-1 would be an effective or even preferential strategy by which to cure DLBCL deserves further investigation in larger cohorts with longer-term follow-up. In addition, the baseline/post-treatment biopsy samples, serum samples and peripheral blood samples were not stored routinely for all patients. More profound studies with paired tumor samples before and after both CD19 CAR-T monotherapy and combination therapy that compare different clinical responsiveness would facilitate the identification of critical factors and pathways that underling the synergistic antitumor effect of anti–PD-1 and the mechanism of CD19 CAR-T resistance.

In conclusion, the combination of CD19 CAR-T cells and nivolumab is safe for further prospective clinical trials in refractory/relapsed B-NHL. Multiple clinical trials investigating the combination of CD19 CAR-T cells and anti-PD-1 or anti-PD-L1 antibodies are ongoing (15). Other strategies, including CAR-T cells engineered to secrete anti-PD-1/PD-L1 antibodies, the engineering of PD-1, knocking down PD-1 expression, and knocking out PD-1, are also currently being evaluated (5, 21). Moreover, systemic comparative analysis of the gene profile of inhibitory pathway mediators in the tumor setting will promote the understanding of the exhaustion mechanism tumors employ and will reveal new therapeutic targets.

## Data Availability

The datasets generated for this study are available on request to the corresponding author.

## Ethics Statement

This study was carried out in accordance with the recommendations of Ethics Committee of New Technology/New Treatment Project of Tianjin First Center Hospital with written informed consent from all subjects. All subjects gave written informed consent in accordance with the Declaration of Helsinki. The protocol was approved by the Ethics Committee of New Technology/New Treatment Project of Tianjin First Center Hospital.

## Author Contributions

MZ designed the research. YC, WL, RS, XJ, LC, XH, LW, TY, and CL performed the research. YC, RS, XJ, WL, and XH analyzed the data. YC and WL wrote the manuscript. YC, WL, and MZ revised the manuscript. All authors approved the final version of the manuscript.

### Conflict of Interest Statement

The authors declare that the research was conducted in the absence of any commercial or financial relationships that could be construed as a potential conflict of interest.
